# Integrated Analysis of Metabolome and Transcriptome Reveals the Difference in Flavonoid Biosynthesis between the Red- and White-Sarcocarp Pomelo Fruits

**DOI:** 10.3390/metabo12121161

**Published:** 2022-11-23

**Authors:** Chenxu Zhao, Jiajia Wang, Yuxia Li, Lei Zhang, Ghazala Nawaz, Shaoyuan Wu, Tao Xu

**Affiliations:** 1 Jiangsu Key Laboratory of Phylogenomics and Comparative Genomics, Jiangsu Joint International Center of Genomics, School of Life Sciences, Jiangsu Normal University, Xuzhou 221116, China; 2 Department of Botany, Kohat University of Science and Technology, Kohat 26000, Pakistan

**Keywords:** pomelo, transcriptome, metabolome, flavonoid, biosynthetic difference

## Abstract

Flavonoids are bioactive secondary metabolites that play multiple roles in plants. However, studies on the flavonoid accumulation of the pomelo fruit are rare. In this study, we conducted a widely targeted metabolome analysis by using ultra-performance liquid chromatography and tandem mass spectrometry and identified 550 metabolites in the sarcocarp from red (*C. maxima* Merr. var. Tubtim Siam) and white pomelos (*C. maxima* (Burm.) Osbeck). A total of 263 significantly changed metabolites were detected from the 550 metabolites. Content analysis of the significantly changed metabolites (SCMs) showed that 138 SCMs were highly accumulated, whereas 125 SCMs were observed with lower content in red-sarcocarp pomelo. Importantly, 103 of the 263 SCMs were flavonoids, including 34 flavonoids, 29 flavonols, 18 flavonoid carbonosides, 9 dihydroflavones, 6 isoflavones, 5 anthocyanins, 1 dihydroflavonol, and 1 chalcone. Gene ontology analysis indicated that upregulated genes in red-sarcocarp pomelo were significantly enriched in GO terms related to flavonoids including flavonoid biosynthetic processes. Several important differentially expressed genes were detected in the correlation network, especially Cg2g009540 which is an orthologous gene of *AtCHS*, also detected in flavonoid biosynthesis networks, and which could be related to the high level of total flavonoids in the red-sarcocarp pomelo. Our study demonstrated the fluctuation of flavonoid biosynthesis in the two pomelo cultivars and laid a theoretical foundation for pomelo breeding to generate fruits with a high flavonoid content.

## 1. Introduction

Flavonoids are ubiquitous secondary metabolites with diverse roles in plants. Flavonoids constitute a class of aromatic bioactive compounds, including sub-classes of flavones, flavonols, isoflavonoids, and anthocyanins. Studies have found that flavonoids are involved in the interactions between the plant and the environment including stress response, color formation of the plant organ, and so on [1,2,3,4]. Furthermore, flavonoids have also been demonstrated to have antioxidant and anti-inflammatory effects in animals [5,6,7]. Dietary flavonoids are important for reverse cholesterol transport, HDL metabolism, and function [8]. As a result, flavonoids have become one of the most attractive metabolites in plants.

With the increase in knowledge in the field of flavonoid metabolism in plants, the unraveling of complexities in the regulation of flavonoid biosynthetic pathways has opened a hotspot for plant scientists. Biosynthesis of flavonoids is a significant event in plant life because it determines the bio-accumulation and structure of bioactive compounds, which greatly affect the physiological activities in plants, particularly citrus fruits. A recent review has summarized the biosynthesis pathways, cell localization, and factors affecting the biosynthesis of flavonoids [9]. In general, it is speculated that citrus-derived flavonoid biosynthesis pathways occur through the phenylpropanoid pathway. Initially, phenylalanine is transformed into p-coumaroyl-CoA with the catalysis of phenylalanine ammonia-lyase, cinnamate 4-hydroxylase, and 4-coumarate-CoA ligase. Then, chalcone synthase (CHS) catalyzes malonyl-CoA and p-coumaroyl-CoA to form chalcone, which transforms into naringenin under the control of chalcone isomerase (CHI). Naringenin is a key intermediate metabolite in the biosynthetic pathway of flavonoids and further converts into flavanones, anthocyanins, flavones, and flavanols [10]. Flavanones are transformed from naringenin. Under the catalysis of flavone synthase (FNS), naringenin is transformed into flavones. Meanwhile, dihydro-flavonols, which play vital roles in the formation of other flavonoids (such as flavonols and anthocyanins), are formed by flavanones under the regulation of flavanone 3-hydroxylase. Several external and internal factors, including transcription factors (TFs), precursors, intermediate enzymes, UV, and root zone salinity, affect the regulatory pathway of the flavonoid pathway [11,12,13].

Citruses are the most widely grown and popular fruits because of their palatable taste and nutritional benefits for human health. Citrus fruits are highly rich in bioactive compounds, including ascorbic acid, phenolic compounds, carotenoids, and flavonoids [14]. Pomelo (*Citrus grandis* (L.) Osbeck) belongs to the genus Citrus of the rutaceae family and is one of the most widely cultivated fruits in the world [15,16]. In China, the pomelo (such as *C. grandis* ‘Tomentosa’) can be used to produce traditional Chinese medicine to relieve several symptoms, such as coughs and phlegm [17,18]. Pomelo contains diverse flavonoids, such as narirutin, naringin, hesperidin, and neo-hesperidin [19,20]. Therefore, the variation of flavonoid profiles in different pomelo cultivars must be studied. In the present study, the total metabolites of two cultivars (*Citrus maxima* (Burm.) Osbeck and *Citrus maxima* Merr. var. Tubtim Siam) were studied using ultra-performance liquid chromatography and tandem mass spectrometry (UPLC-MS/MS) and high-throughput transcriptomic technologies to analyze the biosynthesis regulation and accumulation of flavonoids between pomelo cultivars and their influences on the qualities of pomelo.

## 2. Materials and Methods

### 2.1. Plant Materials

Two pomelo cultivars, comprising white- (*Citrus maxima* (Burm.) Osbeck) and red-sarcocarp pomelos (*Citrus maxima* Merr. var. Tubtim Siam), were cultivated in orchards in Xishuangbanna, Yunnan, China. An approval to collect the plant samples was acquired. Formal identification of the plant material was undertaken by Chunfen Xiao, Hui Teng, and Li Wang from Xishuangbanna Tropical Botanical Garden, Chinese Academy of Sciences (CAS). Voucher specimens of these pomelo cultivars were deposited in the herbarium of Xishuangbanna Tropical Botanical Garden, CAS (Deposition No. 136042 and 162222). Healthy ripe fruits of uniform size were collected in August 2019. Sarcocarp samples at the equatorial plate (0.5 cm thickness) of every five globose fruits were collected, mixed, frozen in liquid nitrogen, and stored at -80 °C in a refrigerator for subsequent analysis. Three biological repeats were carried out for the experiments in this study. Experimental research and studies on the plants complied with relevant institutional, national, and international guidelines and legislation.

### 2.2. UPLC and Electrospray Ionization-Triple Quadrupole Linear Ion Trap (ESI-Q TRAP)-MS/MS

The pulp samples were freeze-dried in a lyophilizer (Scientz-100F) and ground to powder by a grinding machine (MM 400; Retsch, Germany) for 1.5 min at 30 Hz. A total of 100 mg of the powder was dissolved in 0.6 mL 70% aqueous methanol. The solution was kept at 4 °C overnight and then centrifuged at 10,000× *g* for 10 min before the supernatant was collected. A CNWBOND Carbon-GCB SPE Cartridge (250 mg, 3 mL; ANPEL, Shanghai, China) and SCAA-104 (0.22 µm pore size; ANPEL, Shanghai, China) were applied to absorb and filter the extracts for further analysis of total metabolites on UPLC-MS/MS, respectively [21].

The filtrates were analyzed by a UPLC-ESI-MS/MS system (UPLC, Shim-pack UFLC SHIMADZU CBM30A system, Kyoto, Japan; MS, Applied Biosystems 4500 Q TRAP, Waltham, MA, USA). Further, UPLC analysis was performed with a Waters ACQUITY UPLC HSS T3 C18 chromatographic column (1.8 µm, 2.1 mm × 100 mm). The solvent for mobile phase A consisted of ultrapure water with 0.04% acetic acid, and that for mobile phase B was acetonitrile with 0.04% acetic acid. A 0.35 mL/min sample flow rate was used, and the oven temperature was 40 °C. The elution gradient of A:B was 95% A and 5% B at 0 min. Within the first 10 min, B was linearly increased to 95% and held at 95% for 1 min. The proportion of B fell to 5% from 11.00 min to 11.10 min and then remained at 5% for 2.90 min. Then, the effluent was alternatively channeled into an ESI-QTRAP-MS [22].

An API 4500 Q TRAP UPLC/MS/MS system equipped with a linear ion trap (LIT), a triple quadrupole (QQQ), and an ESI turbo IS (ion-spray) interface was operated in controlled mode with positive and negative ions via Analyst 1.6.3. The temperature of ESI was maintained at 550 °C, the MS voltage (or IS voltage) was 5500 V, curtain gas was set at 30 psi, and ion source gases I and II were maintained at 50 and 60 psi, respectively. The parameters of collision-activated dissociation were high. The mass calibration and equipment tuning were carried out with different concentrations of polypropylene glycol, such as 10 µmol/L in the QQQ model and 100 µmol/L in the LIT model. The scans of the QQQ were obtained from the multiple reaction monitoring (MRM) model using nitrogen as the collision gas set at 5 psi. Based on optimized declustering potential and collision energy, each ion pair was scanned following a method described earlier [23].

### 2.3. RNA-Seq Analysis

For transcriptomic analysis of pomelo fruits, total RNA was extracted with the RNAprep pure plant plus kit (Tiangen, Beijing, China) as previously described [24]. The extracted total RNA was checked on a Nano Photometer^®^ spectrophotometer (IMPLEN, CA, USA) and an Agilent 2100 bio analyzer (Agilent Technologies, Clara, CA, USA) to analyze the purity and quality of the RNA, respectively. The degree of stability of the RNA was measured by running the RNA on 1% agarose gel electrophoresis. The cDNA library for each sample was constructed using the NEBNext UltraTM RNA Library Prep Kit (New England Biolabs, NEB, Ipswich, MA, USA), and was sequenced with an Illumina novaseq 6000 platform to generate 150 bp paired-end reads (Illumina, San Diego, CA, USA).

RNA-Seq analysis was performed as previously described [25]. Firstly, clean data for each sample were obtained from the raw data using Trimmomatic (version 0. 39) with default parameters. Secondly, the obtained clean data were mapped to the *C. maxima* genome (v1.0) using STAR (version 2.7.1a) under the 2-pass mapping mode [26,27]. Thirdly, the read counts mapped to each gene were calculated using featureCounts (v2.0.1), and the FPKM values for each gene were obtained using RSEM (v1.3.1). A gene was considered expressed if its average expression level in FPKM was lower than 1. Finally, the DEseq2 package was employed to perform DEG analysis between white- and red-sarcocarp pomelo [28]. Genes with |log2FoldChange| ≥ 2 and adjusted *p*-value ≤ 0.01 were considered to be significantly differentially expressed.

The GO annotation of pomelo genes was assigned using the Trinotate pipeline with default parameters, and was used to build the OrgDb database [29]. The functional enrichment analysis of the above identified DEGs was performed using clusterProfiler at adjusted *p*-values < 0.05 [30].

### 2.4. Gene Expression Analysis by Quantitative Real-Time Polymerase Chain Reaction (qRT-PCR)

The expression of 3 DEGs which encoded CHS, FNS II (flavone synthase II), and SHT (shikimate O-hydroxycinnamoyl transferase) was analyzed by qRT-PCR. The total RNA was extracted from pomelo fruits by using OminiPlant RNA Kit (Kangwei, Taizhou, China). cDNA was synthesized from the total RNA using a reverse transcription kit (TaKaRa, Dalian, China) as previously described [31,32]. β-actin was used as the internal reference gene [26]. Online software Primer 3 (http://bioinfo.ut.ee/primer3-0.4.0/, accessed on 4 November 2022) was used to design qRT-PCR primers (Table S11), and TB Green^TM^ Premix Ex Taq^TM^ II (TaKaRa, Shiga, Japan) was used to perform qRT-PCR analysis on the qPCR instrument (Analytik Jena, Jena, Germany) as described in a previous study [33,34]. The experiments were performed with three repetitions, and the expression was calculated with the 2^−ΔΔCt^ method [35].

### 2.5. Statistical Analysis

Principal component analysis (PCA) and orthogonal partial least squares discriminant analysis (OPLS–DA) were used to simplify the high-dimensional data obtained from various experiments. The MRM of the QQQ was used to quantify metabolites. The core program in R was used to calculate the Pearson correlation coefficient (PCC) between the DEGs and significantly changed metabolites (SCMs). The DEGs and SCMs (|PCC| > 0.9) were selected to draw a correlation network diagram using the software Cytoscape. Significance analysis was performed by using *t*-test in the qRT-PCR experiment.

## 3. Results

### 3.1. PCA and OPLS–DA Analysis in the Fruit of White and Red Pomelo Cultivars

**Figure 1:**
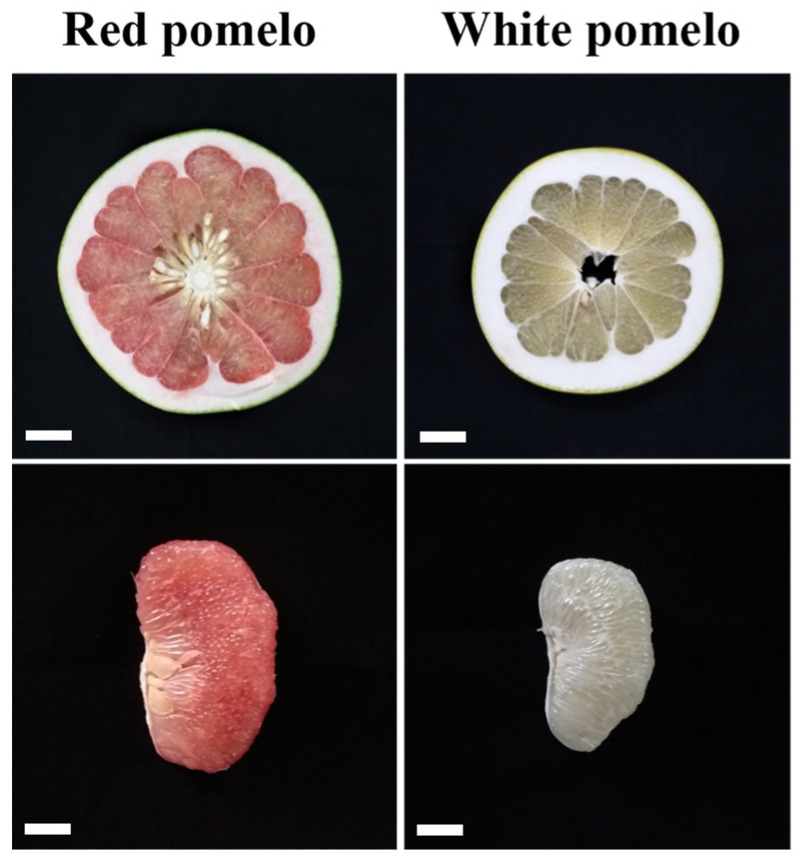
Phenotype of two pomelo cultivars: white- (*Citrus maxima* (Burm.) Osbeck) and red-sarcocarp pomelos (*Citrus maxima* Merr. var. Tubtim Siam). Bars indicate 3 cm.

**Figure 2:**
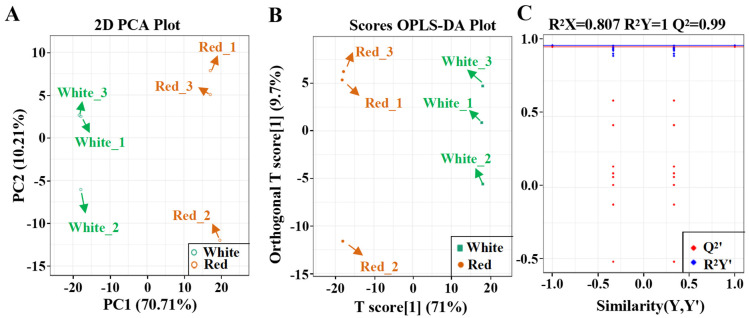
PCA and OPLS-DA on metabolites of pomelo fruit. (**A**) PCA score plot. (**B**) OPLS–DA score plot. (**C**) OPLS–DA permutation analysis model validation graph.

Two pomelo fruits with white and red sarcocarp were collected to compare their flavonoid biosynthesis (Figure 1). To gain insights into the significantly changed flavonoids (SCFs) between the red- and white-sarcocarp pomelos, we performed UPLC–MS/MS assay on the pulp of the pomelos with red or white sarcocarp and detected about 550 metabolites in total (Table S1 (Supplementary Materials)). PCA, a tool that analyzes a small number of principal components to show the structure of multiple variables, was used to analyze the metabolic profile difference between the two pomelo cultivars. The plots for white and red sarcocarp were distributed on the left and right sides, respectively (Figure 2A), implying that significant differences in the metabolic profiles existed between red and white pomelo sarcocarps at principal component 1 (70.71%).

We used OPLS-DA to exclude the small-correlation variables and to achieve the maximum significant differences of the SCMs between the two cultivars. The OPLS–DA score plot results displayed a distinct metabolic difference between the red- and white-sarcocarp cultivars (Figure 2B). A total of 200 alignment experiments were conducted to further verify the OPLS–DA model (R^2^X = 0.807, R^2^Y = 1, and Q^2^ = 0.99). The blue and red lines denote the R^2^Y and Q^2^ of the original model, respectively. The blue and red dots correspond to the R^2^Y’ and Q^2^’ after replacement, respectively. The results indicate that the OPLS-DA model was reliable, and that the SCMs can be selected based on variable importance in projection (VIP) (Figure 2C).

### 3.2. Screening of Significantly Changed Flavonoids

**Figure 3:**
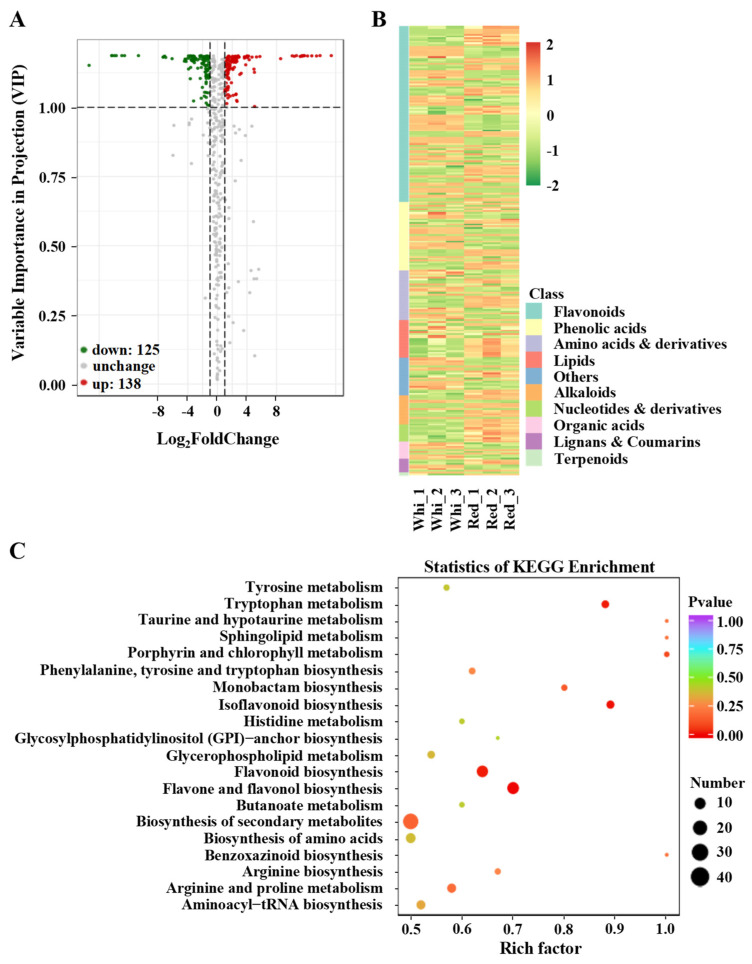
Analysis of SCMs in pomelo fruits. (**A**) Volcano plot of metabolites. (**B**) Heatmap of SCM expression. (**C**) KEGG enrichment analysis of SCMs.

The SCMs in the two cultivars were screened using fold change (FC, ≥2 or ≤0.5) and VIP value (VIP ≥ 1) as the threshold. Among the 550 metabolites, 263 were SCMs between the white- and red-sarcocarp pomelos. A total of 52.5% SCMs (138 in 263) had a higher content, and the rest had a lower content in the red-sarcocarp pomelo compared with those in the white-sarcocarp pomelo (Figure 3A). To further analyze the composition of the SCMs with the greatest difference in content, the absolute log2FoldChange analysis displayed the top 20 SCMs. As shown in Figure S1, 12 SCMs were flavonoids, only 3 of which were found to be highly accumulated in the red-sarcocarp pomelo including 1 anthocyanin and 2 flavonoids. The remaining nine SCMs with lower content in the red-sarcocarp pomelo consisted of three flavonoid carbonosides, two flavonoids, two flavonols, one dihydroflavonol, and one anthocyanin (Figure S1).

To fully comprehend the type and abundance of metabolites present in the two cultivars, we generated a heatmap of 263 SCMs, and the results showed that 103 SCMs were flavonoids, including 34 flavonoids, 29 flavonols, 18 flavonoid carbonosides, 9 dihydro-flavones, 6 isoflavones, 5 anthocyanins, 1 dihydroflavonol, and 1 chalcone (Figure 3B; Table S1). Among these SCFs, 49 exhibited higher levels in the red-sarcocarp pomelo compared with the white-sarcocarp pomelo. Then, the SCMs were annotated to the Kyoto Encyclopedia of Genes and Genomes (KEGG) database to analyze their metabolic pathways [36]. Meanwhile, several detected metabolites that were not annotated in KEGG pathways were added to the corresponding pathways based on their structure. The results of the KEGG enrichment indicated that the SCMs of the two cultivars were significantly enriched in the flavonoid, isoflavonoid, flavone and flavonol biosynthesis pathways (Figure 3C).

### 3.3. Analysis of DEGs and Gene Ontology (GO) Enrichment in Red- and White-Sarcocarp Pomelo

RNA libraries were constructed and sequenced by using the Illumina platform to investigate gene expressions in white- and red-sarcocarp pomelo. After filtering the raw data, checking the sequencing error rate, and inspecting the GC content distribution, approximately 44.91 Gb (149.96 million paired-end reads) of clean bases were obtained, ranging from 6.02 to 9.76 Gb per library (Table S2). The clean data of each library were then mapped to the *C. maxima* genome with an average mapping rate of 98.67% [26]. The read counts and FPKM values for all the annotated genes were calculated. A total of 14,158 genes were found to be expressed in our transcriptome datasets, accounting for 47.00% of all genes (14158/30123) in the *C. maxima* genome [26]. The similarity of the six samples were further assessed by using the above expressed genes. The results showed that the three biological replicates for each cultivar were highly correlated (Figure S2).

**Figure 4:**
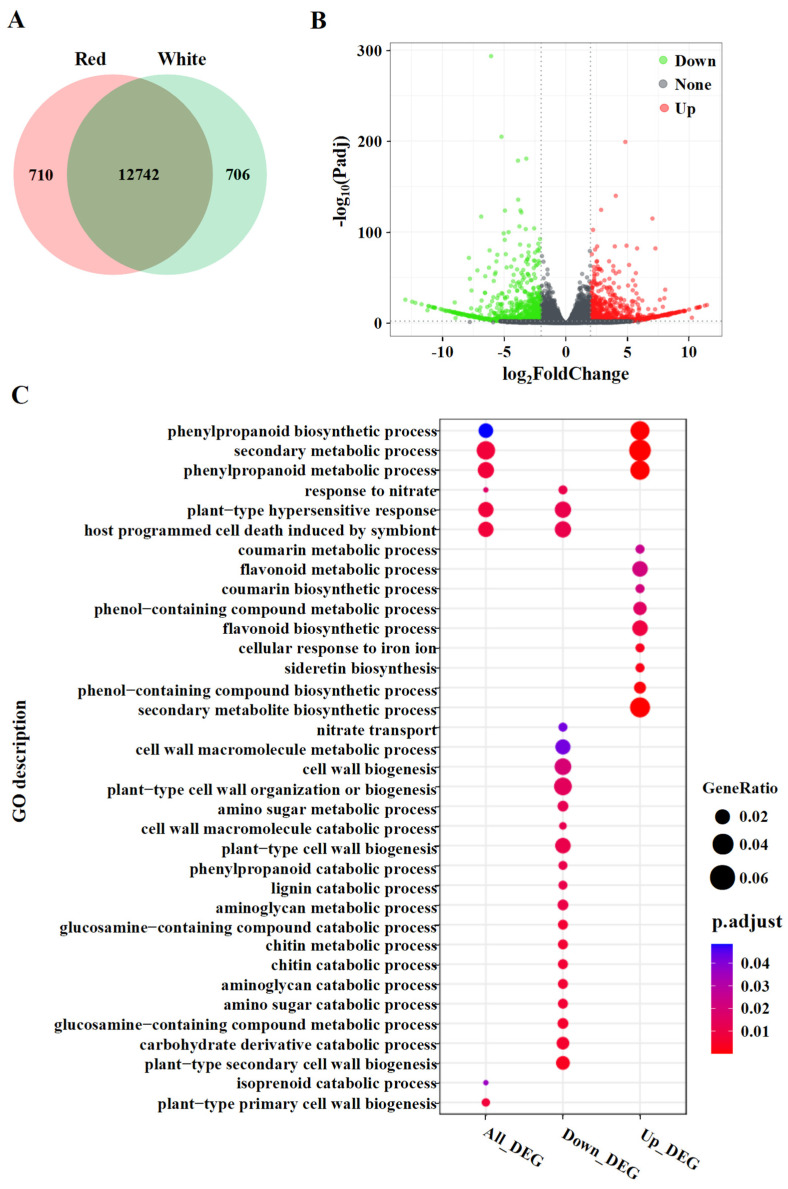
Gene expression analysis and GO annotation of DEGs in pomelo fruits. (**A**) Venn diagram of genes. (**B**) Volcano plot of genes. (**C**) GO annotation of DEGs.

To obtain the significantly differential expression of genes between the two cultivars, we analyzed gene expressions by using the DESeq2 package [28]. A Venn diagram showed that 12,742 genes were expressed both in the white and red pomelo cultivars, whereas 710 and 706 genes were expressed specifically in red- and white-sarcocarp pomelo, respectively (Figure 4A). In the white- vs. red-sarcocarp pomelo comparison, 1593 differently expressed genes (DEGs) were detected based on FC (|log2FoldChange| ≥2) and adjusted *p*-value (≤0.01), in which the expressions of 735 genes were upregulated, and those of 858 genes were significantly downregulated (Figure 4B; Table S3).

To understand the role of TFs involved in the metabolism of pomelo, we annotated 95 DEGs in 31 TF families via iTAK (1.7a) (Table S4). A total of 17 DEGs were detected in the MYB (11 DEGs) and bHLH (6 DEGs) TF families, which are related to flavonoid biosynthesis. Among these DEGs, four MYBs and three bHLHs showed higher expression in the red pomelo than the white pomelo (Table S5).

GO enrichment analysis was performed to categorize the biological functions of the DEGs. For all the DEGs, a total of 35 GO terms were enriched, including 3 for cellular component, 24 for molecular function, and 8 for biological process (Figure 4C; Table S6). For example, GO terms related to “secondary metabolic process” and “phenylpropanoid biosynthetic process” were enriched. To better understand the biological functions of the DEGs, enrichment analysis was conducted for the up- and downregulated DEGs, respectively (Figure 4C, Table S6). By comparing with the white-sarcocarp pomelo, upregulated DEGs in the red-sarcocarp pomelo were significantly enriched in GO terms related to the “phenylpropanoid biosynthetic process” and the “flavonoid biosynthetic process”, etc. The downregulated DEGs were mainly over-represented with GO terms related to “cell wall biogenesis”. These findings were consistent with the characteristics of the red-sarcocarp pomelo, which are enriched in flavonoid metabolites.

### 3.4. Correlation Network Analysis of DEGs and SCMs Related to Flavonoid Biosynthesis

**Figure 5:**
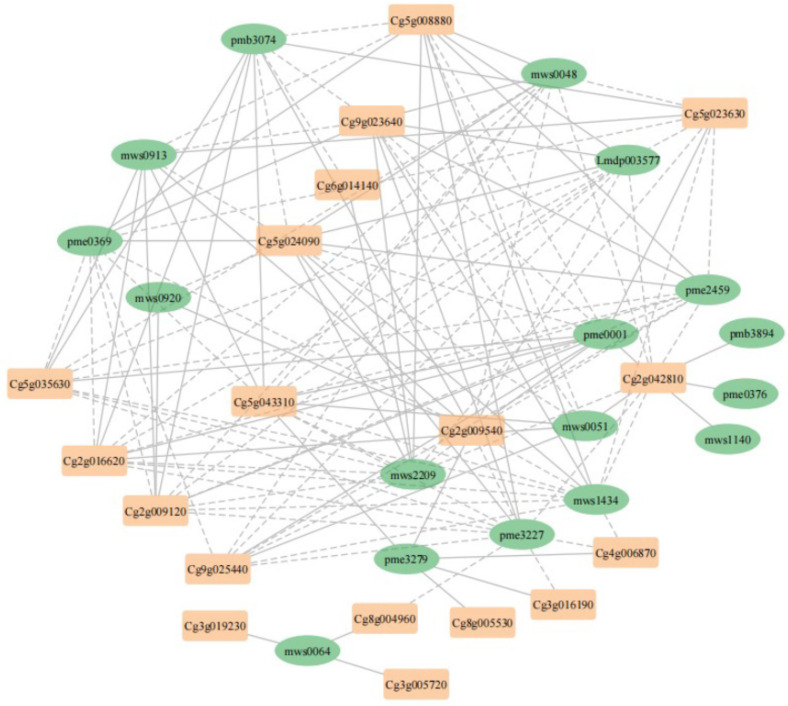
Correlation network of DEGs and SCMs related to flavonoid biosynthesis. Green ellipse, SCMs; light orange squares, DEGs; solid line, positive correlation; dashed line, negative correlation. Lmdp003577: Genistein 7–O–Glucoside; mws0048: Vitexin; mws0051: Acacetin; mws0913: Trifolin; mws1434: Isovitexin; mws2209: Kaempferol-3-O-glucoside; pmb3074: 3–O–p–Coumaroyl quinic acid; pme0001: Neohesperidin; pme0369: Kaempferol–3–O–rutinoside; pme2459: Luteolin–7–O–glucoside; pme3227: Vitexin 2″–O–β–L–rhamnoside; pme3279: 2′-Hydroxygenistein; mws0064: Eriodictyol; mws0920: Tricetin; mws1140: Naringenin chalcone; pmb3894: Di–O–methylquercetin; pme0376: Naringenin. Cg5g043310: *CmBEN1*; Cg5g035630: *CmbHLH42*; Cg2g016620: *Cm5MAT*; Cg2g009120: *CmTT12*; Cg4g006870: *CmCYP711A1*; Cg2g009540: *CmCHS*; Cg3g019230: *CmTT10*.

To study the relationship between genes and metabolites in flavonoid biosynthesis, correlation network analysis was conducted. The 19 DEGs and 17 SCMs related to flavonoid biosynthesis were selected to calculate the PCC, and based on the PCC (|PCC| > 0.9), a correlation network including 18 DEGs and 17 SCMs was drawn (Figure 5; Table S7). In this network, 11 DEGs and 12 SCMs were highly correlated with 5 or more SCMs and DEGs, respectively (Figure 5; Table S8). In the network, Cg2g009540, an orthologous gene of *AtCHS*, was highly related to as many as 12 SCMs. Cg5g035630, highly related to 10 SCMs, could be an orthologous gene of *AtbHLH42*. Cg5g043310, involved in regulation of brassinosteroid metabolism, was related to 12 SCMs, while the other plant hormone-related gene Cg8g004960, involved in ABA transport, was only related to 2 SCMs. Cg4g006870, an orthologous gene of *AtCYP711A1*, was only highly related to three SCMs in the network. The above results indicated the complexity of the flavonoid biosynthetic regulatory network.

### 3.5. Analysis of Flavonoid Biosynthesis Network

**Figure 6:**
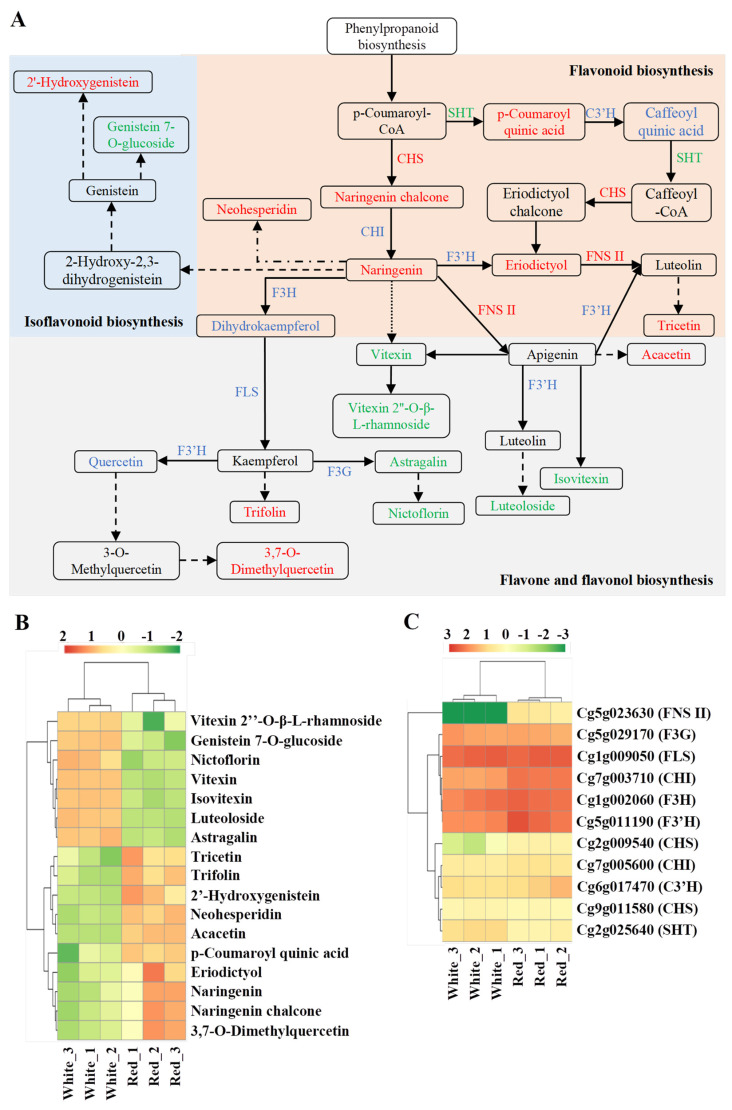
Pathway analysis of flavonoid biosynthesis. (**A**) Flavonoid biosynthesis pathway in pomelo. Solid arrow: molecular interaction or relation; dashed arrow, no genes were detected; dotted arrow, no metabolites were detected; point-line arrow, no genes or metabolites were detected. CHI: chalcone isomerase; CHS: chalcone synthase; C3′H: 5–O–(4-coumaroyl)–D–quinate/shikimate 3′–hydroxylase; F3G: flavonol 3–O–glucosyltransferase; F3H: flavanone 3–hydroxylase; F3′H: flavonoid 3′–hydroxylase; FLS: flavonol synthase; FNS II: flavone synthase II; SHT: shikimate O–hydroxycinnamoyl transferase. (**B**) Heatmap of SCMs in flavonoid biosynthesis. (**C**) Heatmap of gene expressions in flavonoid biosynthesis.

The flavonoid biosynthesis network was analyzed and graphically illustrated based on the analysis of KEGG pathway annotation (Figure 6A). A total of 17 SCMs and 11 relevant genes were detected in the network (Tables S9 and S10). The levels of SCM contents and gene expressions are shown in Figure 6B,C, respectively. The content of 10 metabolites in red-sarcocarp pomelo were higher than those in white-sarcocarp pomelo (Figure 6B). Among 11 genes, only three DEGs were detected: two upregulated genes Cg2g009540 and Cg5g023630, and one downregulated gene Cg2g025640, which encoded CHS (chalcone synthase), FNS II and SHT, respectively (Figure 6C). However, among substrate content, enzyme gene expression and product content, they were not perfectly matched with each other in this network. In general, these results suggested the complexity of the regulatory network in flavonoid biosynthesis.

### 3.6. qRT-PCR Analysis

To confirm the gene expression data obtained from RNA-seq, the transcript levels of the three DEGs in the flavonoid biosynthesis network which encoded *CHS* (Cg2g009540), *FNS II* (Cg5g023630), and *SHT* (Cg2g025640) were analyzed by qRT-PCR using gene-specific primers in the pomelo samples. Comparing with white-sarcocarp pomelo, red-sarcocarp pomelo expressed significantly high levels of *CHS* and *FNS II* and a low level of *SHT* (Figure S3). The expressions of all the DEGs from the qPCR experiment were consistent with those from RNA-seq.

## 4. Discussion

Members of Citrus are globally known for their nutritional and medicinal benefits. However, the presence of flavonoids in different Citrus cultivars and their roles in flavonoid biosynthesis are largely unknown. In this study, widely targeted metabolome and transcriptome sequencing were employed to investigate the differences in flavonoid accumulation between the two pomelo cultivars (*Citrus maxima* (Burm.) Osbeck and *Citrus maxima* Merr. var. Tubtim Siam). The metabolome data suggested that almost half of the metabolites (263 in 550) detected were differentially accumulated between the white-and red-sarcocarp pomelos (Table S1). Flavonoids were the top SCMs between the two cultivars, implying that flavonoids showed the greatest variation and the most important metabolites in pomelo. The differences in metabolites and transcripts of the flavonoid pathway affect petal color in *Nelumbo nucifera* [4]. Recent studies indicated that flavonoids enhance plant tolerance to abiotic stresses [37,38]. The involvement of flavonoids in symbiotic nitrogen fixation in Rhizobia [39] has been investigated. Altogether, our results suggested that flavonoids were one of the most important bioactive components and their abundance might affect characteristic differences of pomelo fruits.

Transcriptome sequencing was carried out to investigate the DEGs in two pomelo cultivars with white and red sarcocarp. GO terms related to flavonoid biosynthesis including “phenylpropanoid biosynthetic process” and “flavonoid biosynthetic process” were found in upregulated DEGs. TFs, such as the MYB, bHLH, and WD40 families, played important roles in flavonoid biosynthetic regulation by forming the MYB-bHLH-WD repeat complex [40]. Overexpression of the *MYB* gene was an important approach to obtain the high content of flavonoids in tobacco [41]. In *Arabidopsis* seedlings, pigment production depended on Transparent Testa Glabra 1 (TTG1, a WD40 TF) and bHLH [42]. In this study, 11 DEGs in the MYB and 6 DEGs in the bHLH family were annotated. Among them, four MYBs and three bHLHs were highly expressed (Table S5). The function of these transcription factors needs to be demonstrated in further studies.

To understand the mechanism of flavonoid biosynthesis in pomelo, we evaluated the PCC between DEGs and SCMs. In the correlation network, Cg5g035630 encoding a TF in the bHLH family was detected, which could participate in the transcriptional regulation of pomelo flavonoid biosynthesis. Phytohormone and stress can also affect flavonoid biosynthesis [43,44,45]. In the network, the detection of plant hormone-related genes (Cg5g043310 and Cg8g004960) and a defense-associated gene (Cg5g024090) indicated potential regulation of plant hormone and stress in flavonoid biosynthesis. *AtCYP711A1* was related to 11 genes (*CHS, CHI,* etc.) in flavonoid biosynthesis [46]. We detected that an orthologous gene of *AtCYP711A1* in pomelo (Cg4g006870) was highly related to three SCMs. *CmMAX1* would be an entry point to further investigate flavonoid biosynthetic regulation in pomelo.

Naringenin was a pivotal intermediate product which could be converted into other flavonoids [10]. As the results suggested in Figure 6, the higher expression of the *CHS* gene (Cg2g009540) could lead to a higher content of naringenin chalcone, and then naringenin was highly accumulated in red-sarcocarp pomelo due to the increase in substrate content, which could be a reasonable interpretation of the higher eriodictyol content and even the high level of total flavonoid in red-sarcocarp pomelo. Naringenin can be converted into 8-C-glucosylnaringenin and then form vitexin, all the steps occurring without regulation by enzymes. Interestingly, we found vitexin content was lower in red-sarcocarp pomelo. In general, the production of apigenin from naringenin was catalyzed by FNS (FNS I and FNS II) [47]. A study in 2019 also found that overexpression of *ZmFNS I* and *ZmFNS II* in *Arabidopsis* plants resulted in apigenin formation and remitted damage caused by UV-B [48]. However, only *FNS II* was detected to be occasionally involved in apigenin formation [49]. In our study, the process of apigenin formation might be catalyzed only by FNS II because just the *FNS II* gene (Cg5g023630) was detected in red-sarcocarp pomelo. Given that only the substrate content had changed significantly, we also speculated that saturation of enzyme activity restricted the synthesis of caffeoyl quinic acid and dihydrokaempferol. Additionally, enzyme genes and direct precursors were not detected; further exploration needs to be conducted on the formation of several flavonoids including 2’-hydroxygenistein, trifolin, and tricetin.

## 5. Conclusions

Metabolome analysis based on UPLC-MS/MS and transcriptome was performed in red- and white-sarcocarp pomelo fruits. DEG analysis revealed that flavonoids were the SCMs between the two kinds of pomelo fruits. GO enrichment analysis showed that specific DEGs were involved in flavonoid biosynthesis-related processes, including the phenylpropanoid biosynthetic process and the flavonoid biosynthetic process. The increase in the *CHS* gene (Cg2g009540) expression could be related to the high level of total flavonoid in red-sarcocarp pomelo. Conclusively, the combined analysis of SCMs and DEGs provided molecular evidence for the flavonoid biosynthesis between red- and white-sarcocarp pomelo and laid a theoretical foundation for the breeding of flavonoid-rich pomelo.

## Data Availability

The datasets generated and analyzed during the current study are available in the NCBI database repository (Public on 1 February 2021, Accession No. GSE162849, https://www.ncbi.nlm.nih.gov/geo/query/acc.cgi?acc=GSE162849, accessed on 4 November 2022).

## Supplementary Materials

The following supporting information can be downloaded at: https://www.mdpi.com/article/10.3390/metabo12121161/s1, Figure S1: Foldchange of top 20 SCMs. SCFs are marked by red boxes, Figure S2: Inter-sample correlation diagram, Figure S3: qRT-PCR analysis of candidate DEGs related to flavonoid biosynthesis, Table S1: List of metabolites in two pomelo cultivars, Table S2: Summary of sequenced RNA libraries, Table S3: List of DEGs in two pomelo cultivars, Table S4: Transcription factor annotation, Table S5: Statistics of transcription factor annotation results, Table S6: GO terms of all the DEGs, Table S7: Pearson correlation coefficient between DEGs and SCMs related to flavonoid biosynthesis, Table S8: Correlation network of DEGs and SCMs related to flavonoid biosynthesis, Table S9: SCMs detected in flavonoid biosynthesis based on KEGG pathway annotation, Table S10: Genes related to SCMs in flavonoid biosynthesis, Table S11: Primers used in qRT-PCR analysis. Citrus β-actin was selected as internal reference. * *p* < 0.05, ** *p* < 0.01, *** *p* < 0.001.
